# Supplementary material for: Analysis of chi angle distributions in free amino acids via multiplet fitting of proton scalar couplings
Source: Magn Reson (Gott). 2024 Aug 19;5(2):103–20. doi: 10.5194/mr-5-103-2024 (PMC11570886; doi:10.5194/mr-5-103-2024)
Supplement: The supplement related to this article is available online at: https://doi.org/10.5194/mr-5-103-2024-supplement. [file mr-5-103-2024-supplement.zip › Syed2024/data/fit1d_fitnmr_output.tar.gz!/fit1d/ile/500/bmse000041_simulation_1_nmredata/1H/sparse_1d.pdf]

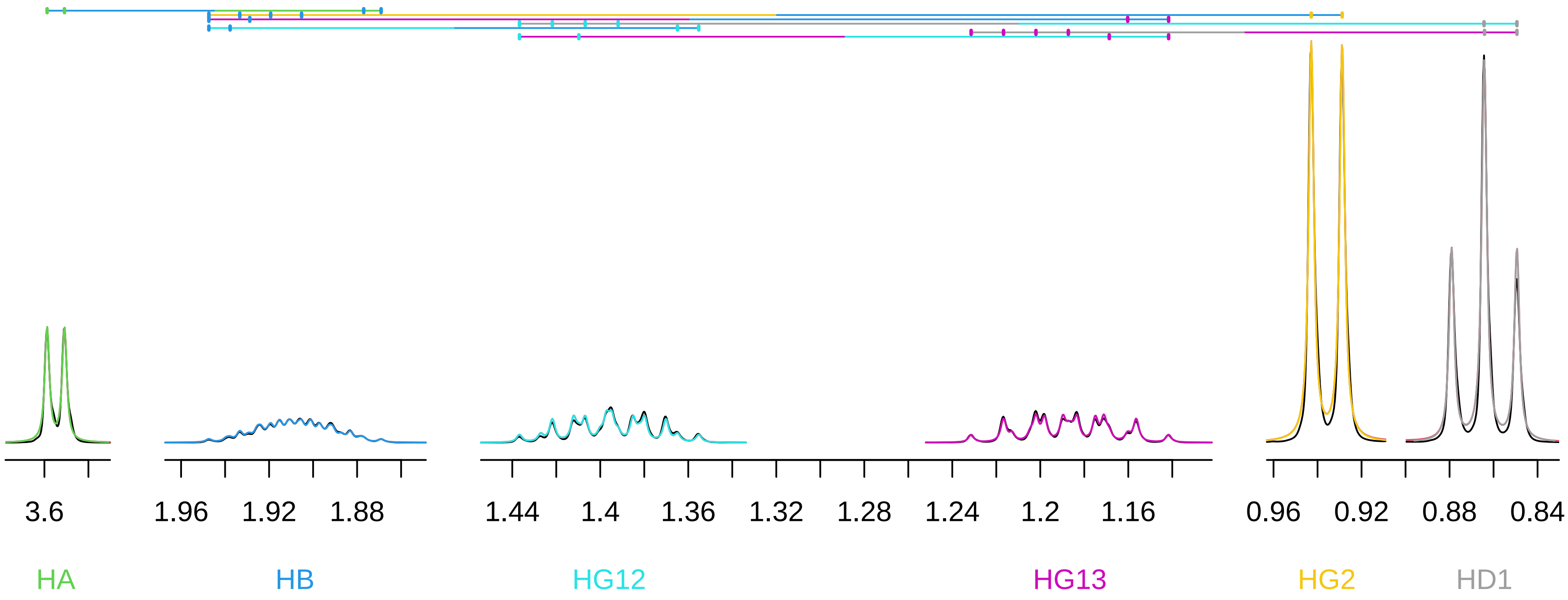

3.6
1.96
1.92
1.88
1.44
1.4
1.36
1.32
1.28
1.24
1.2
1.16
0.96
0.92
0.88
0.84
HA
HB
HG12
HG13
HG2
HD1
